# Dietary Patterns in Relation to Metabolic Syndrome among Adults in Poland: A Cross-Sectional Study

**DOI:** 10.3390/nu9121366

**Published:** 2017-12-17

**Authors:** Edyta Suliga, Dorota Kozieł, Elżbieta Cieśla, Dorota Rębak, Stanisław Głuszek

**Affiliations:** 1 The Department of the Prevention of Alimentary Tract Diseases, The Institute of Nursing and Midwifery, Faculty of Medicine and Health Sciences, Jan Kochanowski University, ul. IX Wieków Kielc 19, 25-317 Kielce, Poland; 2 The Department of Surgery and Surgical Nursing with the Scientific Research Laboratory, The Institute of Medical Sciences, Faculty of Medicine and Health Sciences, Jan Kochanowski University, ul. IX Wieków Kielc 19, 25-317 Kielce, Poland; dorota.koziel@wp.pl (D.K.); dorota.rebak@ujk.edu.pl (D.R.); sgluszek@wp.pl (S.G.); 3 The Department of Developmental Age Research, Institute of Public Health, Faculty of Medicine and Health Sciences, Jan Kochanowski University, ul. IX Wieków Kielc 19, 25-317 Kielce, Poland; elaciesla@poczta.onet.pl

**Keywords:** dietary patterns, adults, metabolic syndrome, International Diabetes Federation/2009

## Abstract

In several populations the associations between diet and the risk of metabolic syndrome have not been fully examined yet. The aim of the study is to identify the main dietary patterns among Polish adults and the evaluation of the relationships of these patterns with metabolic syndrome and its components. The study was conducted on a group of 7997 participants, aged between 37 and 66 years old. Dietary patterns were identified by factor analysis. Metabolic syndrome was defined according to the International Diabetes Federation. Three dietary patterns were identified and designated as: “Healthy”, “Westernized” and “Traditional-carbohydrate”. In the adjusted model, a higher score in the “Westernized” pattern aligns with a higher risk of abnormal glucose concentration (*p*_trend_ = 0.000), but with a lower risk of abnormal High-Density Lipoprotein Cholesterol HDL-cholesterol concentration (*p*_trend_ = 0.024). Higher scores in the “Traditional-carbohydrate” pattern were connected with the risk of abdominal obesity (*p*_trend_ = 0.001) and increased triglycerides concentration (*p*_trend_ = 0.050). Our results suggest that adherence to the “Traditional-carbohydrate” dietary pattern, characterized by higher intakes of refined grains, potatoes, sugar and sweets is associated with a higher risk of abdominal obesity and triglyceridemia. A “Westernized” dietary pattern on the other hand, is related to hyperglycemia. The study results can be used for community-based health promotion and intervention programs to prevent or better manage chronic diseases.

## 1. Introduction

Metabolic syndrome (MetS) is a growing public health concern worldwide. MetS is associated with a higher risk of Type 2 diabetes and cardiovascular morbidity and mortality [1,2,3,4,5]. It is also related to a higher rate of total cancer mortality [6]. In the Polish population, MetS was identified in 30.7% of men and 26.8% of women, according to the International Diabetes Federation (IDF) definition [7].

Study results conducted in different populations indicate that a healthy lifestyle and a balanced diet in particular is critical for preventing or delaying the onset of MetS [8,9,10,11,12,13]. The dietary habits of adult Poles significantly differ from medical recommendations [14,15]. The intake of saturated fatty acids, protein and sugar is too high in the Polish diet [14]. Only 16% of Polish subjects met the World Health Organization (WHO) recommendation for polyunsaturated fats intake.

Intake of vitamins and minerals which are essential for preventing cardiovascular disease, is also inadequate [15]. According to the HDI (Healthy Diet Indicator), a diet quality assessment revealed that the recommended quality of a diet (5–7 points) was found only in 15% of Polish adults, while most of the respondents (about 60%) had a low quality diet (0–3 points).

A standard approach for evaluating the relationships between diet and the risk of chronic disease now involves analyzing of dietary patterns [16]. The approach allows us to achieve a more complex and accurate description of a diet and a more comprehensive illustration of its complexity when compared to studies about the intake of individual nutrients or food items.

However, there have been only a few papers published showing present dietary patterns in the Polish population [17]. The majority of these papers were conducted in small groups [18,19,20]. Despite the growing rate of MetS risk factors in our country [7,21,22], the relationship between diet and metabolic syndrome has not yet been fully examined. Therefore, the aim of the study is to identify the main dietary patterns among Polish adults and analyze the association of these patterns with metabolic syndrome and its components.

## 2. Subjects and Methods

### 2.1. Study Design and Sample Collection

The research material was collected from September 2010 to October 2012 within the framework of the PONS (Polish-Norwegian Study) project. The study was aimed at observing the health status of inhabitants from the south-eastern part of Poland. The project involved 13,172 individuals altogether (8725 women), aged 37–66 years old. Detailed information regarding the project, group selection and research procedures were described in previously published papers [18,23].

Briefly: All men and women aged between 45 and 64 years, who were permanently residing in the Kielecki Region in Poland, were invited to take part in the study. The voluntary participation rate in this age group was 12%. A small number of older people volunteered to take part in the study (65–66 years), and younger (37–44 years), when compared to previously intended age groups. They comprised 0.66% of all participants and were included in the analysis.

Among the participants who volunteered to take part in the study (*N* = 13172), the following were excluded: individuals with cardiovascular disease, stroke, cancer and a history of Type 2 diabetes or missing data concerning the abovementioned diseases (*N* = 3144) Additionally, volunteers lacking socio-demographic data (*N* = 181), data concerning metabolic syndrome components (*N* = 53), smoking and physical activity (*N* = 84), and dietary information (*N* = 1713) were also excluded. Finally, the data of 7997 individuals were included in further analysis.

### 2.2. Ethical Approval

The study was approved by the Ethics Committee from the Cancer Centre and Institute of Oncology in Warsaw and by the Committee on Bioethics at the Faculty of Health Sciences, Jan Kochanowski University in Kielce, Poland (data analysis) (No. 45/2016).

### 2.3. Anthropometric and Blood Pressure Measurements

Trained researchers using standard protocols and techniques measured the participants’ height, weight and waist circumference [24]. Height was measured using a stadiometer, with an accuracy of 0.1 cm. Waist circumference was measured halfway between the lower rib edge and the upper iliac crest using a metric measure with an accuracy of 0.1 cm. The measurements were conducted using SECA equipment (SECA GmbH & Co., Hamburg, Germany).

Weight was measured by using a body composition analyzer (Tanita SC 240MA, Tanita Corp., Tokyo, Japan), with an accuracy of 0.1 kg. Blood pressure (BP) was measured by using the blood pressure monitor Omron (Model M3 Intellisense, Mannheim, Germany). Blood pressure was measured on the artery of the right upper limb when the participant was seated. In the study, an average of two measurements were used for analysis.

### 2.4. Biochemical Profiling

Trained nurses obtained blood samples from the antecubital vein of subjects who had fasted for at least 10 h. The samples were collected in ten different laboratories. The samples were labeled with the unique participant number and then placed in an insulated box containing several chilled packs, which reliably kept the internal box temperature at 4 °C. The boxes were transported to the Swietokrzyskie Cancer Centre (the biobank location) where the samples were extracted and placed in freezers.

Concentrations of fasting plasma glucose, triglycerides (TG), and HDL were measured in the hospital’s laboratory, which met the standards of the National Reference laboratory. The concentration of HDL-cholesterol was measured using the colorimetric non-precipitation method. The glucose concentration in the blood serum was measured using the enzyme method with hexokinase while the concentration of triglycerides (TG) was assessed using the phosphogliceride oxidaseperoxidase method.

### 2.5. Dietary Assessment and Food Pattern Derivation

Food consumption data were collected by administering the Food Frequency Questionnaire (FFQ). The PONS FFQ was constructed based on a previously-developed and validated FFQ for the Poland branch of the PURE study. FFQ has good validity and is reproducible in relation to the referential method [25].

FFQ consisted of a list of 67 standard size products. The study participants were asked about how frequently they consumed certain portions of each product during the last year. The frequencies of consumption were classified as follows: “6 times a day or more, 4–5 times a day, 2–3 times a day, once a day, 5–6 times a week, 2–4 times a week, once a week, 1–3 times a month, less frequently than once a month or not at all, I don’t know, I refuse to answer the question”.

In order to determine dietary patterns, food items and beverages from the questionnaire were combined into 33 food groups. The reason for placing a given product in a group was to identify its nutrient profile, culinary usage and groups used in other studies [26]. Some food items were analyzed separately because the profile of their nutrients was unique (e.g., margarine, eggs). The method for combining products and beverages in groups is described in Supplementary Table S1.

### 2.6. Socio-Demographic Variables and Lifestyle Data

The socio-demographic variables included: Age (continuous variable), place of residence (urban; rural), education (university; lower than university), marital status (married or in a stable relationship; single or a widow). The respondents who smoked cigarettes on a daily basis during the study were classified as current smokers while those who had not smoked for longer than 6 months were classified as former smokers and the rest were regarded as nonsmokers. Physical activity was evaluated with the use of the International Physical Activity Questionnaire (IPAQ)—the long form [27]. Total physical activity was expressed in minutes/day.

### 2.7. Definition of Terms

The prevalence of metabolic syndrome was defined using recommendations from the International Diabetes Federation Task Force on Epidemiology and Prevention (joint interim statement in 2009) [28]. According to the established definition, MetS was identified in persons who met at least three out of five criteria: waist circumference ≥ 94 cm in males and ≥80 cm in females; fasting glucose ≥ 100 mg/dL (5.5 mmol/L) or diabetes treatment; HDL cholesterol ≤ 40 mg/dL (1.0 mmol/L) in males and ≤50 mg/dL (1.3 mmol/L) in females or drug treatment for reduced HDL cholesterol; TG ≥ 150 mg/dL (1.7 mmol/L) or drug treatment for elevated TG; and systolic BP ≥ 130 mmHg or diastolic BP ≥ 85 mmHg, or drug treatment for hypertension.

A trained interviewer (a nurse) using face-to-face interviews of the subjects during the last 30 days collected data about medication that lower arterial blood pressure, the level of cholesterol and triglycerides as well as data about antidiabetic medication.

### 2.8. Statistical Analyses

In order to identify dietary patterns factor analysis was used. The FFQ answers about the frequency and portion intake of certain food items provided the basis for defining certain variables in the analysis. The data obtained this way was transformed into daily dietary doses and then normalized using the Z-score procedure. Both the Kaiser-Meyer-Olkin index (0.742) and Bartlett’s test (*p* < 0.001) showed that the correlation among the variables was sufficiently strong for factor analysis. The applied procedure and analysis excluded products which did not show any relationship with other food items or had a weak correlation with them. These included: low-fat milk, hard cheese, tea, coffee, low-calorie fizzy drinks, fruit juice, margarine, butter, lard, bigos (a stew made of sauerkraut and/or fresh cabbage, meat and mushrooms), boiled vegetables with mayonnaise, potato and cheese dumplings, chips, and chicken. The Varimax procedure was used to rotate these factors and, thereby improve the interpretation of the results.

We established the number of the determined factors using Kaiser’s criterion (>1). It was additionally verified through Cattell’s criterion (scree plot test). Food items were retained in the pattern if the factor loading value was above 0.30. Finally, three factors were derived, which altogether accounted for 26.7% of the whole variance. The derived factors (dietary patterns) were labeled on the basis of our data interpretation and on earlier literature. The values were calculated for each dietary factor. In the range of each pattern, the division of subjects into quartile groups was carried out. Additionally, the subject features were divided into quartile groups based on the range of dietary patterns. Further statistical analyses were done in the quartiles of each dietary factor.

For the categorical data: gender, place of residence, education, marital status, smoking, and the metabolic syndrome components including: abdominal obesity, elevated BP, increased glucose and TG concentration, decreased HDL concentration and the structure indicators were calculated. The associations between structure indicators for each factor were analyzed by using the Chi-square test.

The arithmetic mean, standard deviation and median were calculated for age, body mass index (BMI) and physical activity in the quartiles of each factor (dietary pattern). The significance of the differences between the averages was calculated by using one factor analysis of variance ANOVA or Kruskal-Wallis one-way analysis of variance, depending on the characteristic distribution and variance homogeneity. Inter-group differences were evaluated by using the Bonferroni post-hoc test (Table S2–S7). To assess the relationship between dietary patterns and the risk of MetS and its components, a logistic regression analysis was applied and the odds ratios (OR) indicator and 95% confidence intervals (CI) were calculated. Two models were presented. In the first model crude data was considered while in the second model, the risk of the inaccurate values for MetS features was adjusted to the gender, place of living, education, marital status, smoking, total physical activity, age and BMI (continuous variables). For the adjusted model, confounding principles were selected on the basis of the correlation matrix between the variables chosen for analysis. The basis for the derivation included a significant value of the correlation coefficient between a dependent and independent variable and weak or faint correlations between dependent variables were the basis for their derivation. All *p*-values presented are 2-tailed; *p* < 0.05 was considered significant. The statistical analysis was carried out with the use of Statistical Package for the Social Science (SPSS)^®^ software, version 16.0 (IBM, Armonk, New York, NY, USA).

## 3. Results

The most frequently consumed foods among the participants were: refined grains, fruit, vegetables, sugar and sweets (Table S8). Fish was found to be usually consumed once a week, but processed meat was consumed much more often at 6.4 times a week. Three major dietary patterns were extracted by means of factor analysis: “Healthy, Westernized, and “Traditional-carbohydrate”.

Pattern I—“Healthy”—which allowed for 10.55% of variance, was positively correlated with the intake of fruit and vegetables, sour cabbage, whole grains, as well as with the consumption of yogurt and cottage cheese (dairy products of low fat content), fish and nuts and negatively correlated with refined grains (Table 1). Pattern II—“Westernized”—was most strongly correlated with the consumption of fried dishes, oil and mayonnaise, red meat, processed meat, eggs, sugar sweetened beverages, alcohol, sugar and sweets. Pattern III—“Traditional-carbohydrate”—was positively correlated with the consumption of potatoes and refined grains as well as the intake of soups, sugar and sweets and high fat milk, and negatively correlated with whole grains.

Obesity (BMI ≥ 30.0 kg/m^2^) was found in 26.5% of the individuals (in 28.6% of the men and in 26.5% of the women), and MetS in 39.9% of the individuals (in 48.1% of the men and in 35.8% of the women). The percentage of obese individuals and with MetS increased with age. Participants with excess body mass were also less physically active when compared to the subjects with normal Body Mass Index.

Participants with higher scores in the “Healthy” pattern more often included women and younger individuals who were more physically active, had higher education, and were residents of cities, and less frequently among current smokers (Table 2). However, no differences in marital status were observed across the quartile. In the lowest quartile of “Healthy”, the smallest number of participants with an abnormal pattern of HDL-cholesterol concentration and the highest number with abnormal glucose concentration were observed. Participants in the next quartiles of this pattern did not show any differences regarding BMI nor the incidence of MetS and its other components.

Participants with a high adherence to a “Westernized” pattern more often involved men, residents of cities, younger individuals and people in stable relationships as well as current and former smokers. The higher the quartile of the “Westernized” pattern, the greater the percentage of participants with an abnormal glucose concentration and low HDL-cholesterol concentration. However, there were no differences with relation to BMI, abdominal obesity, increased BP, abnormal TG concentration and the occurrence of MetS in participants across the quartile of this pattern.

Participants with higher scores in the “Traditional-carbohydrate” pattern more often included men, older individuals, people with a lower education, those in stable relationships, more physically active and current smokers. In the lowest quartile of this pattern there were people more countryside inhabitants than city residents; there were also more former smokers. The higher the score of “Traditional-carbohydrate”, the greater the percent of participants who had an increased TG concentration. There were no significant differences found concerning BMI, incidents of MetS and its other components in individuals across the quartile of this pattern.

In the model, before adjustment, higher scores of the “healthy” pattern were related to a lower risk of abnormal glucose concentration (*p*_trend_ = 0.016), but a higher risk of abnormal HDL-cholesterol concentration (*p*_trend_ = 0.017) (Table 3). Reverse associations were noted in individuals with higher scores in the “Westernized” pattern, in whom the risk of abnormal glucose concentration was higher (*p*_trend_ = 0.000), but the risk of abnormal HDL-cholesterol concentration was lower (*p*_trend_ = 0.000). In the two above-mentioned patterns, there were no differences among the risk of MetS, abdominal obesity, an increased BP and abnormal TG concentration across the quartile. A higher score of “Traditional-carbohydrate” pattern was correlated with a higher risk of BP (*p*_trend_ = 0.020), abnormal glucose (*p*_trend_ = 0.018), TG concentration (*p*_trend_ = 0.000), and MetS (*p*_trend_ = 0.033).

There was a higher risk of abnormal HDL-cholesterol concentration in the model when adjusted for gender, age, education, place of residence, marital status, smoking, physical activity, and BMI in Q3 of the “Healthy” pattern, there was a higher risk of abnormal HDL-cholesterol concentration (Q3 vs. Q1, *p* = 0.017) (Table 4). A higher score of the “Westernized” pattern was related to a greater risk of abnormal glucose concentration (*p*_trend_ = 0.000), but a lower risk of abnormal HDL-cholesterol concentration (*p*_trend_ = 0.024). Higher scores of the “Traditional-carbohydrate” pattern were associated with a risk of abdominal obesity (*p*_trend_ = 0.001) and increased TG concentration (*p*_trend_ = 0.050).

## 4. Discussion

The dietary patterns identified by us were not significantly related to the risk of MetS after adjusting for confounding principles. We only found associations between the dietary patterns and some of the MetS components. It is true that, in several papers, it was shown that Westernized or Western (unhealthy) patterns have been associated with a higher risk of MetS [13,29], the Westernized patterns are characterized by a high consumption of meat or meat products, snacks, sugar-sweetened beverages, and baked desserts, which provide high amounts of saturated fatty acids and simple carbohydrates as added sugars.

However, the results of studies have not unambiguously confirmed such relations. Kang and Kim [30] stated that the Westernized pattern was not associated with MetS or its components in either men or women. Woo et al. [31] showed that the positive association between the prevalence of MetS and the dietary pattern score was found only for men with the meat dietary pattern while the traditional pattern and the snack pattern were not associated with an increased prevalence of MetS. Kimokoti et al. [32] found that none of the clusters were associated with MetS or other MetS components over a mean follow-up of seven years. Unambiguous scores related to the above-mentioned associations may have occurred because dietary patterns vary according to age, ethnicity, culture, and other lifestyle factors.

However, the results of the majority of studies confirm quite consistently that healthy dietary patterns may decrease the risk of central obesity [33,34,35,36], while patterns described as unhealthy (Western) increase this risk [32,33,37,38]. The findings of our study that show an increased risk of abdominal obesity in participants with a higher adherence to the “Traditional-carbohydrate” pattern in their diet. As the results of some studies suggest, the proportion of carbohydrates in the diet was not important in predicting changes in weight or waist circumference [39,40]. The sources of carbohydrates were found to be significant. Fogelholm et al. [40] noted that a high intake of refined (white) bread was related to an increased growth of waist circumference. McKeown et al. [41] showed that higher intakes of refined grains are associated with higher visceral adipose tissue, while Serra-Majem and Bautista-Castaño [42] found that increases in white bread consumption during four years of follow-up were associated with a greater growth of waist circumference (mean waist change of 1.11 cm in the lowest and of 2.39 cm in the highest quartile).

Halkjaer et al. [39] report a positive relationship between foods containing carbohydrates simple sugars or refined grains and the changes in waist circumference in women. The scores concerning the high impact of potato consumption and the risk of obesity, including abdominal obesity, are ambiguous. Heidar-Beni et al. [43] found a positive association between potato consumption and abdominal obesity among 11–13-year-old girls. Borch et al. [44], on the basis of a literature review, stated that the association between potatoes (not including French fries) and adiposity was positive in two studies and was neutral in two other studies. It is possible that the way potatoes are prepared is important. Murakami et al. [45] showed that the dietary glycemic index and glycemic load were independently associated with a higher risk of central obesity (waist circumference ≥ 88 cm in women and ≥102 cm in men). In this study, the positive predictive foods for dietary glycemic index were breads and potatoes, which is in compliance with the results of our analyses.

Several authors have shown that a high intake of carbohydrates may also result in hyperlipidemia [46,47,48]. Min et al. [49], have noted that a high dietary glycemic index and glycemic load is related to an increase in TG concentration. However, total LDL- and HDL-cholesterol levels were not significantly connected with carbohydrate intake, which has been confirmed by the results of our study. This phenomenon has been explained by Flannery et al. [50], who showed that age-induced muscle insulin resistance results in decreased postprandial muscle glycogen synthesis and diverts carbohydrates toward de novo lipid synthesis in the liver. The effect of this phenomenon is called hypertriglyceridemia.

Among the participants with a high score in the “healthy“ pattern, which was correlated with a high intake of fruits, vegetables and low fat dairy products, we found an increased risk of too low HDL concentration. Kent et al. [51] noted that when people move towards a low-fat, plant-based diet, HDL levels decrease while other indicators of cardiovascular risk improve. The results of lifestyle interventions that promote a low-fat and plant-based diet also show lower concentration of HDL-cholesterol [52].

The results of our study have shown that the risk of an abnormal HDL concentration was the smallest in participants with the highest score of the “Westernized” pattern. Yanai et al. [53], on the basis of literature review, have concluded that the role of dietary factors for HDL metabolism remained largely unknown. Mensink et al. [54] found that all types of fatty acid ingestion elevated HDL when substituted for carbohydrates. Recently conducted analyses have also revealed that the consumption of ≥3 servings of red meat/day showed no negative effects on blood lipid, lipoprotein concentrations and blood pressure, but did result in higher HDL concentrations [55]. Thus, the results of our study are in accordance with the above-mentioned analysis. Moreover, the “Westernized” pattern in our study is significantly correlated with higher alcohol consumption. Results obtained by other authors confirm that moderate alcohol consumption (0.1–5.0 g/day) was associated with a low prevalence of MetS and its components, including HDL-cholesterol [56].

A different study conducted in the Polish population has shown that both a medium (15–30 g/day) as well as high (>30 g/day) consumption of alcohol is connected to a smaller risk of a low HDL concentration, by 40% and 44% respectively [57]. The results of our study have also revealed that high adherence to the “westernized” pattern in a diet increased the risk of an elevated glucose concentration. Although these associations were slightly weaker after adjusting for confounding principles, they still remained significant. Several authors have shown that a high consumption of meat, processed meat in particular, can increase the risk of the occurrence of Type 2 diabetes [58]. Ley et al. [59] have confirmed that a higher intake of red meat in women is related to undesirable values of biomarkers of glucose metabolism biomarkers.

Substitution of a serving of red meat with an alternative protein was associated with a healthier biomarker profile of inflammatory and glucose metabolism. The meta-analysis of epidemiological studies concerning the consumption of meat has shown that meat intake is associated with fasting glucose and insulin concentrations in Caucasians without diabetes mellitus [60]. Both processed and unprocessed red meat were associated with a higher fasting glucose after the adjustment for potential confounding principles (not including BMI). Every additional 50-g serving of processed meat per day was associated with a 0.021 mmol/L higher fasting glucose concentration. For every additional 100-g serving of unprocessed red meat per day fasting glucose was 0.037 mmol/L higher. Several authors believe that the results were largely attenuated after the adjustment for BMI, possibly because of the independent effects of BMI on both meat intake and fasting glucose concentrations. However, it should be mentioned that the “Westernized” pattern, identified in our population, is positively correlated with the consumption of sugar sweetened beverages, sugar and sweets, which could constitute an additional cause for a higher risk of abnormal glucose concentration in the participants with high adherence to this pattern.

Our study has several limitations. First of all, because this is a cross-sectional study, the causal link between dietary patterns and MetS development remains unclear. It is possible that the diagnosis of even a single metabolic disorder may, in some people, lead to a change in their dietary habits. The three derived patterns explained about 26.7% of the total variation. This may not explain all Polish dietary patterns thoroughly. Another limitation includes the lack of data concerning body mass reducing diets. However, studies conducted among Polish people have shown that despite problems with excessive body weight, adherence to a low-calorie diet was declared by only about 1% of the respondents [15].

The strong side of this study is the large and ethnically homogenous group of participants. Moreover, in our study we used dietary patterns determined a posteriori, which reflect reality better than the patterns determined on the basis of previously adopted assumptions. They are closer to the real reality, in which food and nutrients are consumed in complex systems, while the lack of preliminary assumptions makes them independent from current knowledge and scientific views.

## 5. Conclusions

In conclusion, we found that the “Traditional-carbohydrate” dietary pattern, characterized by higher intakes of refined grains, sugar, sweets and potatoes, was associated with a higher risk of abdominal obesity and triglyceridemia. The Westernized dietary pattern, characterized by a high consumption of fats, red and processed meat, alcohol, sugar sweetened beverages, sugar and sweets, was related to hyperglycemia, but also to a lower risk of abnormal HDL concentration. In the “Healthy” pattern, associated with a high intake of fruit, vegetables and low fat dairy products, we found a higher likelihood of low HDL-cholesterol concentration.

The results of our analyses show that public health policy should be focused more on cooperation with food producers in order to change the ingredients of some food products (to lower the amount of sugar). It is also essential to introduce an appropriate price policy that will encourage consumers to choose food products beneficial for the prevention of diet-related diseases, especially among consumers with low socio-economic status. Limiting the marketing pressure of food products not recommended for excessive consumption, and developing a system of dietary counseling among local communities will surely be beneficial for public health as well. Health education related to altering eating habits of the general public should be conducted on many levels, including families, schools, work places, health centers, and places of leisure activities.

## Figures and Tables

**Table 1:** Factor-loading matrix for major dietary patterns *.

Food Groups	Factor I	Factor II	Factor III
Vegetables	0.677		
Fruit	0.614		
Cottage cheese	0.485		
Whole grains	0.471		−0.464
Sauerkraut	0.442		
Fish	0.429		
Yogurt	0.400		
Nuts	0.311		
Fried foods		0.555	
Vegetable oils		0.507	
Processed meat		0.469	
Mayonnaise		0.455	
sugar sweetened beverages		0.383	
Eggs		0.381	
Red meat		0.381	
Alcohol		0.348	
Boiled potatoes			0.602
Refined grains	−0.403		0.590
Soup			0.526
Whole milk			0.340
Sugar and sweets			0.335
Percentage of variance explained (%)	10.55	8.14	8.01

* Values < 0.30 were excluded for simplicity.

**Table 2:** The characteristic of the study participants in quartile categories of the dietary patterns ($\overset{-}{X}$, SD; *N*, %). Values above the cutoff were given for MetS components.

Variables	Q_1_ (*N* = 1999)	Q_2_ (*N* = 2000)	Q_3_ (*N* = 1999)	Q_4_ (*N* = 1999)	*p*
	$\overset{-}{X}(\mathrm{SD})/N(\%)$	$\overset{-}{X}(\mathrm{SD})/N(\%)$	$\overset{-}{X}(\mathrm{SD})/N(\%)$	$\overset{-}{X}(\mathrm{SD})/N(\%)$	
**Factor I—“Healthy” Dietary Pattern**					
gender (women)	1118(55.9)	1333(66.6)	1425(71.3)	1473(73.7)	**<0.000 ^A^**
place of living (city)	1111(55.6)	1242(62.1)	1334(66.7)	1347(67.4)	**<0.000 ^A^**
university education	403(20.2)	550(27.5)	633(31.7)	731(36.6)	**<0.000 ^A^**
marital status (married)	1605(80.3)	1593(79.7)	1628(81.4)	1603(80.4)	0.540 **^A^**
former smokers	662(33.1)	667(33.4)	691(34.6)	736(36.8)	**<0.000 ^A^**
current smokers	495(24.8)	388(19.4)	342(17.1)	316(15.8)	
age (years)	54.57(5.43)^4^	54.89(5.32)	54.95(5.35)	54.06(5.32) ^1^	**0.028 ^B^**
body mass index (BMI) (kg/m^2^)	27.71(4.36)	27.7(4.35)	27.73(4.42)	27.78(4.60)	0.956 **^B^**
PA (min/day)	168.2(147.1) ^3,4^	170.9(147.0) ^4^	184.1(148.7) ^1,4^	206.5(151.9) ^1,2,3^	**<0.000 ^B^**
abdominal obesity (cm)	1415(70.8)	1451(72.5)	1434(71.7)	1411(70.6)	0.489 **^A^**
elevated BP * (mmHg)	1383(69.2)	1397(69.8)	1408(70.4)	1346(67.3)	0.165 **^A^**
glucose (mg/dL)	628(31.4)	567(28.3)	555(27.8)	559(28.0)	**0.036 ^A^**
HDL (mg/dL)	421(21.1)	490(24.5)	511(25.6)	482(24.1)	**0.006 ^A^**
TG (mg/dL)	612(30.6)	600(30.0)	594(29.7)	597(29.9)	0.931 **^A^**
MetS	804(40.2)	799(40.1)	802(39.3)	785(39.9)	0.928 **^A^**
**Factor II—“Westernized” Dietary Pattern**					
gender (women)	1573(78.7)	1430(71.5)	1300(65.0)	1046(52.3)	**<0.000 ^A^**
place of living (city)	1252(62.6)	1206(60.3)	1228(61.4)	1348(67.4)	**<0.000 ^A^**
university education	569(28.5)	582(29.1)	570(28.5)	596(29.8)	0.763 **^A^**
marital status (married)	1526(76.3)	1586(79.3)	1630(81.5)	1687(84.4)	**<0.000 ^A^**
former smokers	664(32.2)	691(34.5)	693(34.7)	728(36.4)	**<0.000 ^A^**
current smokers	259(13.0)	348(17.4)	409(20.5)	525(26.3)	
age (years)	55.90(5.26) ^2,3,4^	55.06(5.26) ^1,4^	54.61(5.33) ^1,4^	53.91(5.38) ^1,2,3^	**<0.000 ^B^**
BMI (kg/m^2^)	27.72(4.53)	27.75(4.40)	27.66(4.360	27.80(4.44)	0.800 **^B^**
PA (min/day)	176.0(140.3) ^3,4^	180.5(148.1) ^4^	188.9(153.8) ^1,4^	184.5(155.0) ^1,2,3^	**0.049 ^B^**
abdominal obesity (cm)	1444(72.2)	1437(71.9)	1430(71.5)	1400(70.0)	0.436 **^A^**
elevated BP * (mmHg)	1390(69.5)	1397(69.8)	1368(68.4)	1379(69.0)	0.778 **^A^**
glucose (mg/dL)	511(25.6)	542(27.1)	578(28.9)	678(33.9)	**<0.000 ^A^**
HDL (mg/dL)	550(27.5)	476(23.8)	451(22.6)	427(21.4)	**<0.000 ^A^**
TG (mg/dL)	628(31.4)	588(29.4)	572(28.6)	615(30.8)	0.202 **^A^**
MetS **	819(41.0)	770(38.5)	773(38.7)	828(41.4)	0.123 **^A^**
**Factor III—“Traditional-Carbohydrate” Dietary Pattern**					
gender (women)	1564(78.2)	1408(70.4)	1297(64.9)	1080(54.0)	**<0.000 ^A^**
place of living (city)	1538(76.9)	1312(65.6)	1175(58.8)	1009(62.9)	**<0.000 ^A^**
university education	836(41.8)	628(31.4)	483(24.2)	370(18.5)	**<0.000 ^A^**
marital status (married)	1498(74.9)	1593(79.7)	1649(82.5)	1689(84.5)	**<0.000 ^A^**
former smokers	794(39.7)	672(33.6)	664(33.2)	626(31.3)	
current smokers	328(16.4)	368(18.4)	387(19.4)	458(22.9)	**<0.000 ^A^**
age (years)	54.70(5.42) ^4^	54.76(5.43)	54.87(5.32)	55.14(31)^1^	**0.043 ^B^**
BMI (kg/m^2^)	27.75(4.41)	27.71(4.47)	27.70(4.35)	27.75(4.49)	0.976 **^B^**
PA (min/day)	159.4(133.2) ^2,3,4^	170.1(143.8) ^3,4^	190.9(153.7) ^1,4^	210.4(161.0) ^1,2,3^	**<0.000 ^B^**
abdominal obesity (cm)	1390(69.5)	1443(72.2)	1440(72.0)	1438(71.9)	0.201 **^A^**
elevated BP * (mmHg)	1338(66.9)	1395(69.8)	1390(69.5)	1411(70.6)	0.072 **^A^**
glucose (mg/dL)	550(27.5)	552(27.6)	602(30.1)	605(30.3)	0.080 **^A^**
HDL (mg/dL)	492(24.6)	482(24.1)	472(23.6)	458(22.9)	0.629 **^A^**
TG (mg/dL)	540(27.0)	593(29.6)	629(31.5)	641(32.1)	**0.002 ^A^**
MetS **	765(38.3)	784(39.2)	818(40.9)	823(41.2)	0.184 **^A^**

SD: standard deviation; Q—quartile; PA—physical activity; BP—blood pressure; TG—triglycerides; MetS—metabolic syndrome; **^A^** Chi-square test; **^B^** Kruskal-Wallis one-way analysis of variance test and Median test; —numbers in **bold** indicate statistically significant results; ^1,2,3,4^—statistically significant results between quartiles—the results of Bonferroni post-hoc test; * elevated BP ≥ 130/85 mmHg; ** MetS—At least three out of five criteria: waist circumference ≥94 cm in males and ≥80 cm in females; fasting glucose ≥ 100 mg/dL (5.5 mmol/L) or diabetes treatment; HDL cholesterol ≤ 40 mg/dL (1.0 mmol/L) in males and ≤50 mg/dL (1.3 mmol/L) in females or drug treatment for reduced HDL cholesterol; TG ≥ 150 mg/dL (1.7 mmol/L) or drug treatment for elevated TG; and systolic BP ≥ 130 mmHg or diastolic BP ≥ 85 mmHg, or drug treatment for hypertension.

**Table 3:** Risk of MetS and its components occurrence in quartile categories of the dietary patterns (Model I—unadjusted) odds ratios (OR) and their 95% confidence intervals (95% CI).

Dietary Patterns		Abdominal Obesity	*p*	Elevated BP *	*p*	Glucose	*p*	HDL	*p*	TG	*p*	MetS **	*p*
I *	Q1	1.0		1.0		1.0		1.0		1.0		1.0	
	Q2	1.09 (0.95–1.25)	0.216	1.03 (0.90–1.18)	0.648	**0.86** **(0.75–0.99)**	**0.034**	**1.22** **(1.05–1.141)**	**0.010**	0.97 (0.85–1.11)	0.672	0.99 (0.87–1.12)	0.862
	Q3	1.05 (0.91–1.20)	0.507	1.06 (0.93–1.21)	0.386	**0.84** **(0.73–0.96)**	**0.011**	**1.29** **(1.11–1.50)**	**0.001**	0.96 (0.87–1.10)	0.535	0.99 (0.88–1.13)	0.949
	Q4	0.99 (0.86–1.13)	0.889	0.92 (0.80–1.05)	0.209	**0.85** **(0.74–0.97)**	**0.017**	**1.19** **(1.03–1.38)**	**0.021**	0.96 (0.84–1.20)	0.606	0.96 (0.84–1.10)	0.539
*P* trend		0.754		0.282		**0.016**		**0.017**		0.580		0.584	
II *	Q1	1.0		1.0		1.0		1.0		1.0		1.0	
	Q2	0.97 (086–1.10)	0.659	0.98 (0.87–1.10)	0.756	**1.00** **(1.003–1.28)**	**0.045**	**0.79** **(0.70–1.90)**	**0.000**	0.89 (0.79–1.002)	0.055	0.90 (0.81–1.01)	0.075
	Q3	0.96 (0.84–1.11)	0.662	0.95 (0.93–1.09)	0.452	**1.18** **(1.03–1.36)**	**0.017**	**0.77** **(0.66–0.89)**	**0.000**	0.87 (0.76–1.002)	0.053	0.91 (0.80–1.03)	0.137
	Q4	0.90 (0.78–1.03)	0.125	0.97 (0.85–1.11)	0.706	**1.49** **(1.30–1.71)**	**0.000**	**0.72** **(0.62–0.83)**	**0.000**	0.97 (0.85–1.11)	0.657	1.02 (0.89–1.16)	0.772
*P* trend		0.126		0.507		**0.000**		**0.000**		0.551		0.756	
III *	Q1	1.0		1.0		1.0		1.0		1.0		1.0	
	Q2	1.13 (0.99–1.30)	0.069	1.14 (0.997–1.30)	0.056	1.00 (0.87–1.15)	0.951	0.97 (0.84–1.12)	0.706	1.14 (0.99–1.30)	0.064	1.04 (0.92–1.18)	0.546
	Q3	1.13 (0.99–1.30)	0.082	1.13 (0.99–1.29)	0.077	1.13 (0.99–1.30)	0.069	1.35 (0.82–1.09)	0.947	**1.24** **(1.08–1.42)**	**0.002**	1.12 (0.98–1.27)	0.087
	Q4	1.20 (0.98–1.29)	0.095	**1.18** **(1.04–1.35)**	**0.013**	**1.14** **(1.00–1.31)**	**0.050**	0.91 (0.79–1.05)	0.207	**1.27** **(1.11–1.46)**	**0.000**	1.13 (0.99–1.28)	0.061
*P* trend		0.117		**0.020**		**0.018**		0.189		**0.000**		**0.033**	

HDL: high-density lipoprotein cholesterol; Q—quartile; *—I—Healthy; II—Westernized; III—Traditional-carbohydrate; —numbers in **bold** indicate statistically significant results; BP—blood pressure; TG—triglycerides; MetS—metabolic syndrome; * elevated BP ≥ 130/85 mmHg; ** MetS—At least three out of five criteria: waist circumference ≥ 94 cm in males and ≥80 cm in females; fasting glucose ≥ 100 mg/dL (5.5 mmol/L) or diabetes treatment; HDL cholesterol ≤ 40 mg/dL (1.0 mmol/L) in males and ≤ 50 mg/dL (1.3 mmol/L) in females or drug treatment for reduced HDL cholesterol; TG ≥ 150 mg/dL (1.7 mmol/L) or drug treatment for elevated TG; and systolic BP ≥ 130 mmHg or diastolic BP ≥ 85 mmHg, or drug treatment for hypertension.

**Table 4:** Risk (OR) of MetS and its components occurrence in quartile categories of the dietary patterns (Model II adjusted for sex, age, place of living, education, marital status, smoking, physical activity and BMI) (OR; 95% CI).

Dietary Patterns		Abdominal Obesity	*p*	Elevated BP *	*p*	Glucose	*p*	HDL	*p*	TG	*p*	MetS **	*p*
I *	Q1	1.0		1.0		1.0		1.0		1.0		1.0	
	Q2	1.03 (0.84–1.26)	0.781	1.06 (0.92–1.23)	0.385	0.93 (0.81–1.08)	0.339	1.15 (0.99–1.35)	0.067	1.02 (0.89–1.17)	0.774	1.03 (0.89–1.19)	0.679
	Q3	1.00 (0.81–1.24)	0.970	1.16 (0.99–1.34)	0.055	0.91 (0.79–1.06)	0.221	1.21 (1.03–1.42)	0.017	1.04 (0.90–1.20)	0.610	1.03 (0.89–1.20)	0.648
	Q4	0.89 (0.72–1.10)	0.274	0.96 (0.82–1.11)	0.580	0.93 (0.80–1.08)	0.337	1.16 (0.98–1.36)	0.071	1.05 (0.90–1.21)	0.529	1.05 (0.90–1.22)	0.537
*P* trend		0.131		0.818		0.520		0.126		0.441		0.560	
II *	Q1	1.0		1.0		1.0		1.0		1.0		1.0	
	Q2	0.89 (0.72–1.10)	0.274	0.96 (0.82–1.11)	0.580	0.93 (0.80–1.1.08)	0.337	1.16 (0.98–1.36)	0.071	1.05 (0.90–1.21)	0.529	1.05 (0.90–1.22)	0.537
	Q3	1.05 (0.87–1.26)	0.545	0.98 (0.86–1.11)	0.752	1.10 (0.97–1.26)	0.134	**0.87** **(0.76–0.99)**	**0.032**	0.89 (0.79–1.01)	0.066	0.90 (0.80–1.02)	0.112
	Q4	1.03 (0.83–1.27)	0.773	1.14 (0.98–1.33)	0.095	**1.35** **(1.16–1.58)**	**0.000**	**0.84** **(0.71–0.98)**	**0.031**	0.91 (0.78–1.05)	0.192	1.04 (089–1.21)	0.627
*P* trend		0.832		0.079		**0.000**		**0.024**		0.967		0.880	
III *	Q1	1.0		1.0		1.0		1.0		1.0		1.0	
	Q2	**1.37** **(1.08–1.64)**	**0.006**	1.13 (0.98–1.30)	0.098	0.93 (0.80–1.07)	0.314	1.00 (0.80–1.16)	0.999	1.10 (0.95–1.27)	0.214	1.01 (0.88–1.17)	0.846
	Q3	**1.27** **(1.03–1.57)**	**0.023**	1.10 (0.95–1.28)	0.190	1.03 (0.88–1.20)	0.725	0.99 (0.85–1.16)	0.910	**1.16** **(1.02–1.35)**	**0.048**	1.06 (0.91–1.23)	0.431
	Q4	**1.55** **(1.24–1.94)**	**0.000**	1.15 (0.98–1.34)	0.092	0.93 (0.80–1.10)	0.415	0.98 (0.83–1.15)	0.770	1.18 (0.99–1.36)	0.055	1.05 (0.90–1.23)	0.514
*P* trend		**0.001**		0.453		0.543		0.958		**0.050**		0.593	

Q—quartile; *—I—Healthy; II—Westernized; III—Traditional-carbohydrate; —numbers in **bold** indicate statistically significant results; BP—blood pressure; TG—triglycerides; MetS—metabolic syndrome; * elevated BP ≥ 130/85 mmHg; ** MetS—At least three out of five criteria: waist circumference ≥ 94 cm in males and ≥80 cm in females; fasting glucose ≥ 100 mg/dL (5.5 mmol/L) or diabetes treatment; HDL cholesterol ≤ 40 mg/dL (1.0 mmol/L) in males and ≤ 50 mg/dL (1.3 mmol/L) in females or drug treatment for reduced HDL cholesterol; TG ≥ 150 mg/dL (1.7 mmol/L) or drug treatment for elevated TG; and systolic BP ≥ 130 mmHg or diastolic BP ≥ 85 mmHg, or drug treatment for hypertension.

## Supplementary Materials

The following are available online at http://www.mdpi.com/2072-6643/9/12/1366/s1, Table S1: Food grouping used in the dietary pattern analyses, Table S2: The results of Bonferroni post-hoc test for age (Factor I), Table S3: The results of Bonferroni post-hoc test for PA (min/day) (Factor I), Table S4: The results of Bonferroni post-hoc test for age (Factor II), Table S5: The results of Bonferroni post-hoc test for PA (min/day) (Factor II), Table S6: The results of Bonferroni post-hoc test for age (Factor III), Table S7: The results of Bonferroni post-hoc test for PA (min/day) (Factor III), Table S8: The most frequently consumed foods (servings/day).
