# Supplementary material for: Dietary Patterns in Relation to Metabolic Syndrome among Adults in Poland: A Cross-Sectional Study
Source: Nutrients. 2017 Dec 17;9(12):1366. doi: 10.3390/nu9121366 (PMC5748816; doi:10.3390/nu9121366)
Supplement: Supplementary file 1 [file nutrients-09-01366-s001.docx]

**Dietary patterns in relation to metabolic syndrome among adults in Poland: a cross-sectional study**

Supplementary file:

Table S1. Food grouping used in the dietary pattern analyses.

| Food groups | Food items |
| --- | --- |
| refined grains | white bread, pasta |
| whole grains | dark bread, corn flakes, groats |
| vegetables | broccoli, carrots, garlic, onions, raw white cabbage, red beetroots, tomatoes, green leafy vegetables |
| sauerkraut | sauerkraut |
| fruit | apples, bananas, oranges, seasonal fruits (berries) |
| nuts | nuts |
| whole milk | whole milk |
| low-fat milk | low-fat milk |
| cottage cheese | cottage cheese |
| yoghurt | yoghurt |
| hard cheese | hard cheese |
| fish | fish |
| chicken | chicken |
| eggs | eggs |
| red meat | pork, beef, liver |
| processed meat | bacon, ham, sausages, frankfurters |
| lard | lard |
| butter | butter |
| vegetable oils | rapeseed, soya, sunflower oil, olive oil, and other oils |
| margarine | margarine |
| mayonnaise | mayonnaise |
| boiled potatoes | boiled potatoes |
| chips | chips |
| bigos | bigos (*a* *stew made of sauerkraut and/or fresh cabbage, meat and mushrooms*) |
| fried foods | fried foods |
| soup | soup |
| sugar and sweets | sugar, chocolate and chocolate-products, candies, cakes, cookies |
| fruit juice | fruit juice |
| diet carbonated soft drinks | diet carbonated soft drinks |
| sugar sweetened beverages | coca cola or other beverages of this type |
| alcoholic beverages | vodka, grape wine, fruit wine, beer |
| coffee | coffee |
| tea | tea |

Table. S2. The results of Bonferroni post-hoc test for age (Factor I).

| Factor I | Q1 | Q2 | Q3 | Q4 |
| --- | --- | --- | --- | --- |
| Q1 | - |  |  |  |
| Q2 | 0.350 | - |  |  |
| Q3 | 0.163 | 1.000 | - |  |
| Q4 | **0.025** | 1.000 | 1.000 | - |

Table. S3. The results of Bonferroni post-hoc test for PA (min/day) (Factor I).

| Factor I | Q1 | Q2 | Q3 | Q4 |
| --- | --- | --- | --- | --- |
| Q1 | - |  |  |  |
| Q2 | 1.000 | - |  |  |
| Q3 | **0.014** | 0.150 | - |  |
| Q4 | **0.000** | **0.000** | **0.000** | - |

Q – quartile; PA – physical activity; numbers in **bold** indicate statistically significant results

Table S4. The results of Bonferroni post-hoc test for age (Factor II).

| Factor II | Q1 | Q2 | Q3 | Q4 |
| --- | --- | --- | --- | --- |
| Q1 | - |  |  |  |
| Q2 | **0.000** | - |  |  |
| Q3 | **0.000** | 0.690 | - |  |
| Q4 | **0.000** | **0.000** | **0.001** | - |

Table S5. The results of Bonferroni post-hoc test for PA (min/day) (Factor II).

| Factor II | Q1 | Q2 | Q3 | Q4 |
| --- | --- | --- | --- | --- |
| Q1 | - |  |  |  |
| Q2 | 1.000 | - |  |  |
| Q3 | **0.014** | 0.150 | - |  |
| Q4 | **0.000** | **0.000** | **0.000** | - |

Q – quartile; PA – physical activity; numbers in **bold** indicate statistically significant results

Table S6. The results of Bonferroni post-hoc test for age (Factor III).

| Factor III | Q1 | Q2 | Q3 | Q4 |
| --- | --- | --- | --- | --- |
| Q1 | - |  |  |  |
| Q2 | 0.058 | - |  |  |
| Q3 | 0.181 | 0.992 | - |  |
| Q4 | **0.042** | 0.815 | 0.934 | - |

Table S7. The results of Bonferroni post-hoc test for PA (min/day) (Factor III).

| Factor III | Q1 | Q2 | Q3 | Q4 |
| --- | --- | --- | --- | --- |
| Q1 | - |  |  |  |
| Q2 | **0.003** | - |  |  |
| Q3 | **0.000** | **0.022** | **-** |  |
| Q4 | **0.000** | **0.000** | **0.000** | - |

Q – quartile; PA – physical activity; numbers in **bold** indicate statistically significant results

Table S8. The most frequently consumed foods (servings/day).

| Food groups | X(SD) | Me(Q1-Q3) |
| --- | --- | --- |
| Alcohol | 0.31(0,54) | 0.11(0.04-0.38) |
| \| Whole milk \| \| --- \| | 0.13(0.33) | 0.00(0.00-0.07) |
| \|  \| \| --- \|   Cottage cheese | 0.26(0.26) | 0.43(0.07-0.43) |
| \| Yogurt \| \| --- \| | 0.41(0.39) | 0.43(0.07-0.43) |
| \| Mayonnaise \| \| --- \| | 0.08(0.14) | 0.07(0.00-0.07) |
| \| Fruit \| \| --- \| | 1.78(1.10) | 1.57(1.06-2.21) |
| \| Sauerkraut \| \| --- \| | 0.14(0.14) | 0.07(0.07-0.14) |
| \| Vegetables \| \| --- \| | 2.08(1.08) | 1.92(1.28-2.63) |
| \| Boiled potatoes \| \| --- \| | 0.55(0.35) | 0.43(0.43-0.79) |
| \| Eggs \| \| --- \| | 0.32(0.27) | 0.43(0.14-0.43) |
| \| Processed meat \| \| --- \| | 1.06(0.78) | 0.92(0.57-1.28) |
| \| Red meat \| \| --- \| | 0.38(0.23) | 0.35(0.21-0.56) |
| \| Fish \| \| --- \| | 0.16(0.13) | 0.14(0.07-0.14) |
| \| Refined grains \| \| --- \| | 1.70(1.64) | 1.14(0.43-2.64) |
| \| Whole grains \| \| --- \| | 1.26(1.29) | 0.92(0.43-2.14) |
| \| Soup \| \| --- \| | 0.56(0.31) | 0.43(0.43-0.79) |
| \| Coca - Cola \| \| --- \| | 0.08(0.29) | 0.00(0.00-0.00) |
| \| Sugar and sweets \| \| --- \| | 3.23(3.15) | 2.28(0.71-4.64) |
| \| Nuts \| \| --- \| | 0.06(0.13) | 0.00(0.00-0.07) |
| \| Fried foods \| \| --- \| | 0.30(0.15) | 0.29(0.29-0.29) |
